# A Case of Acinic Cell Carcinoma Metastasizing to the Heart

**DOI:** 10.1007/s12105-025-01808-9

**Published:** 2025-07-07

**Authors:** Aniqa Nuzhat Chowdhury, Shayna DeSando, Fatima Zahra Aly

**Affiliations:** Wake Forest Baptist Department of Pathology, Wake Forest Baptist Medical School, 1 Medical Boulevard, Winston-Salem, NC 27157 USA

**Keywords:** Acinic cell carcinoma, Salivary gland neoplasm, Metastasis to heart, NOR-1

## Abstract

We present the case of a patient with acinic cell carcinoma of the parotid gland who was found to have metastasis to the left atrium of the heart. The pathologic findings and prior reports of metastasis of acinic cell carcinoma are discussed in this article. Grading of acinic cell carcinoma and utility of immunohistochemical study using NOR-1 is discussed.

## Introduction

Acinic cell carcinoma of the salivary glands is a low-grade malignancy. Acinic cell carcinoma accounts for 17% of primary malignant salivary gland tumors [1] and 1–6% of all epithelial salivary gland neoplasms [2]. The primary cause of treatment failure and death in patients with salivary gland carcinoma is distant metastasis [3]. A study has found that distant metastasis occurs in approximately 20% of cancer cases involving major salivary glands [4]. Distant metastasis has been reported to occur in approximately 12% of cases of acinic cell carcinoma, with the lungs being the most common site [5, 6]. Metastasis of salivary gland carcinoma to the heart is rare, with only seven cases reported in the literature. This case report presents the first published case of metastasis of acinic cell carcinoma of the parotid gland to the left atrium.

## Case Report

**Fig. 1:**
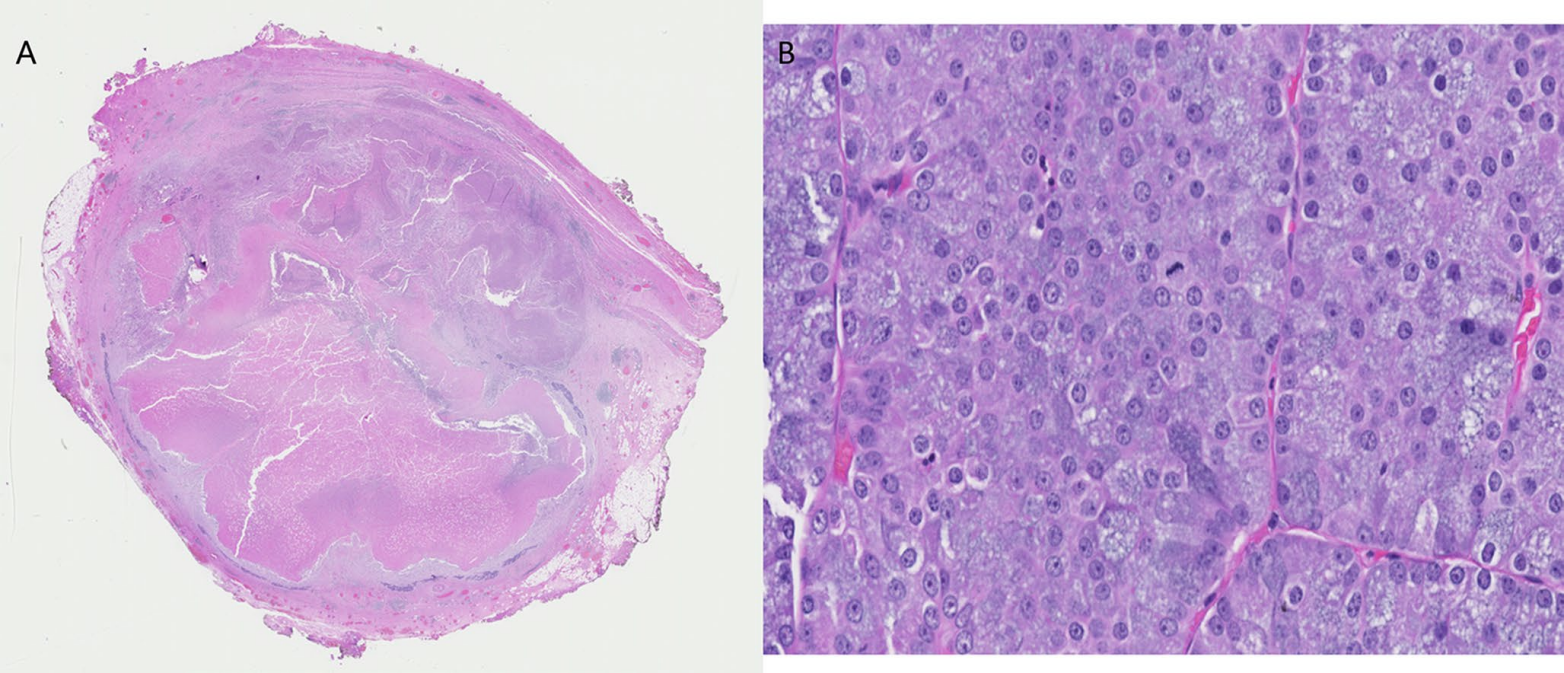
Histology images of original parotid gland tumor: **A**) Low power magnification, **B**) High power magnification

A 56-year-old female was found to have a mass in the parotid gland and subsequently underwent parotidectomy and radical neck dissection in 2015. Histopathology showed multifocal acinic cell carcinoma without lymphovascular or perineural invasion and with close margins of less than 0.1 cm in multiple areas of the right carotid bed. Review of the original parotid gland tumor showed two foci of acinic cell carcinoma (2.8 cm and 1.9 cm in size) with extensive tumor necrosis. The tumor showed large, polyhedral acinar cells with basophilic cytoplasm, prominent nucleoli, and nuclear pleomorphism, morphologically consistent with acinic cell carcinoma (Fig. 1). Margins were negative for carcinoma; however, the distance from the closest margin was less than 0.1 cm at multiple foci. Lymphovascular and perineural invasion were not identified. Following parotidectomy, the patient underwent adjuvant radiation therapy to the right carotid bed (30 fractions over 6 weeks with total dose of 60 Gy).

Three years later, the patient was found to have multiple lung nodules, and biopsy revealed metastatic acinic cell carcinoma with a *BRAF V600E* mutation, which was detected via next generation sequencing. She received 5 fractions of stereotactic body radiotherapy (SBRT) to the dominant left lung nodule (total dose of 50 Gy). Subsequent imaging showed a gradual increase in the size of her multiple lung nodules. Despite the clinical progression of her disease, the patient did not undergo any additional treatment or follow-up until May 2023 (4 years after initial SBRT), when she began experiencing dyspnea on exertion and weight loss. A computed tomography (CT) scan showed further increases in mediastinal adenopathy and lung metastasis.

Due to the worsening disease burden, the patient commenced treatment with a combined BRAF/MET inhibitor (dabrafenib and trametinib). Approximately 2 weeks after beginning this regimen, a CT scan showed findings concerning for either a thrombus in the left atrium and pulmonary veins or progression of her metastatic disease. The patient was treated for thromboembolic disease with anticoagulation. A subsequent cardiac magnetic resonance imaging (MRI) 7 months after beginning treatment with the BRAF/MET inhibitor revealed a 4 cm mass arising from the right pulmonary veins and entering the left atrium, with impingement of the mitral valve (Fig. 1A). The mass was excised, and subsequent pathology from the left atrium mass showed an epithelial neoplasm with polyhedral acinar cells, basophilic cytoplasm, eccentric nuclei, and prominent nucleoli (Fig. 3). The cells from the tumor in the left atrium were less pleomorphic than those from the original parotid gland tumor. Immunohistochemical studies were performed, demonstrating DOG1 (Fig. 4A), MOC31, and Claudin-4 positivity; GATA3 was negative. Periodic Acid-Schiff with diastase (PASD) stain was positive (Fig. 4C). Nuclear expression of NOR-1, which is a specific and sensitive marker for acinic cell carcinoma of the salivary gland [7], showed strong nuclear staining (Fig. 4B), confirming that the metastasis is acinic cell carcinoma. Immunohistochemistry for Ki-67 showed less than 1% staining for both the tumor from the left atrium (Fig. 5A) and the tumor from the fourth ventricle of the brain (Fig. 6B), indicating a low proliferation rate.

**Fig. 2:**
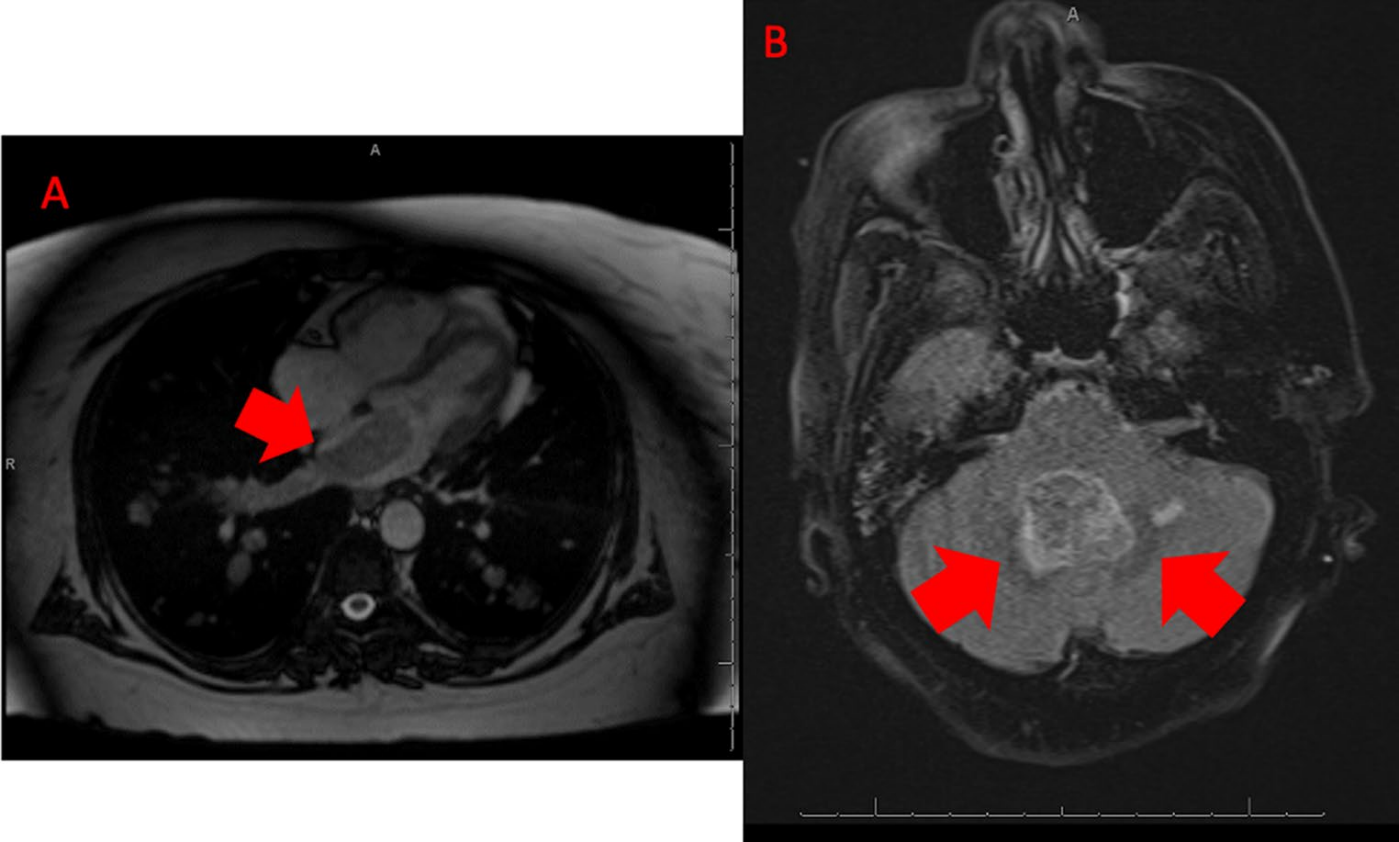
**A**) Cardiac MRI showing tumor indicated by red arrow arising from the pulmonary veins and into the left atrium. **B**) Brain MRI showing pontomedullary hemorrhage as indicated by red arrows

**Fig. 3:**
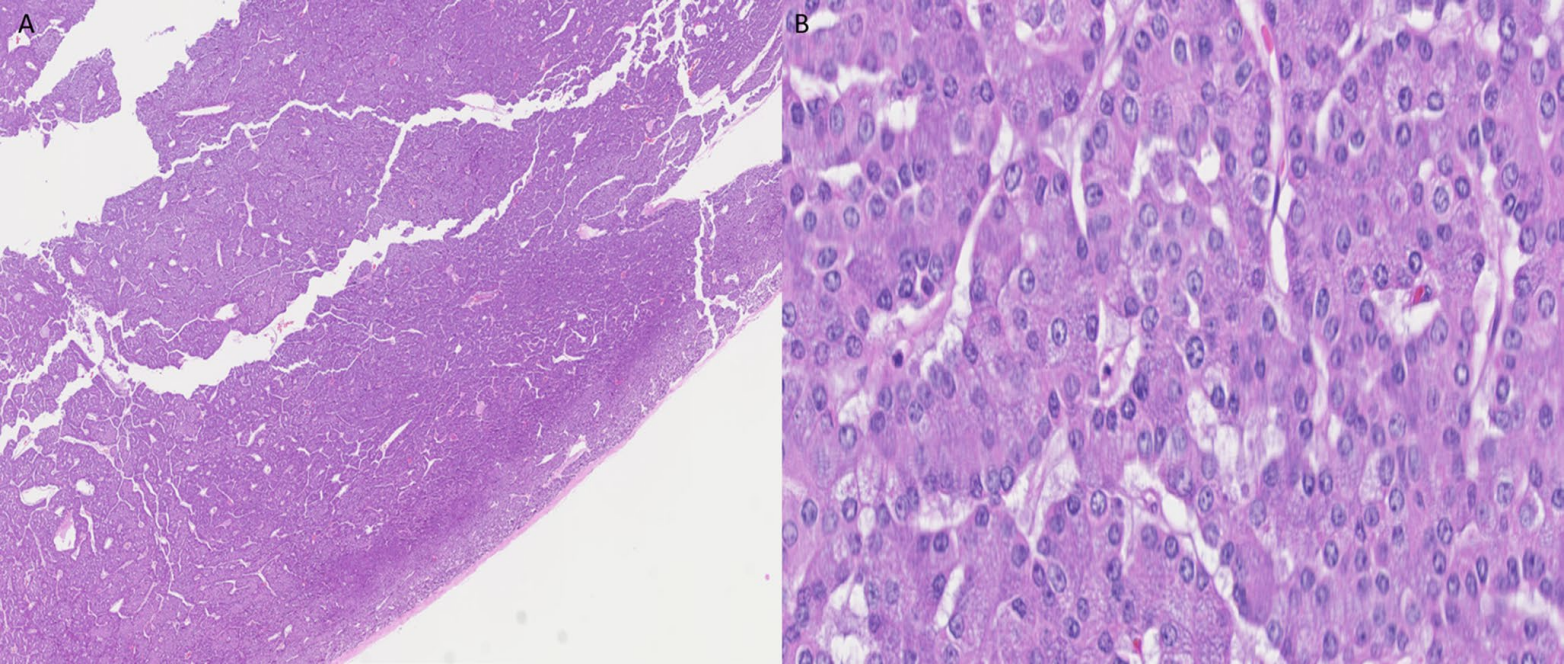
Histology images of tumor from left atrium: **A**) Low power magnification, **B**) High power magnification

**Fig. 4:**
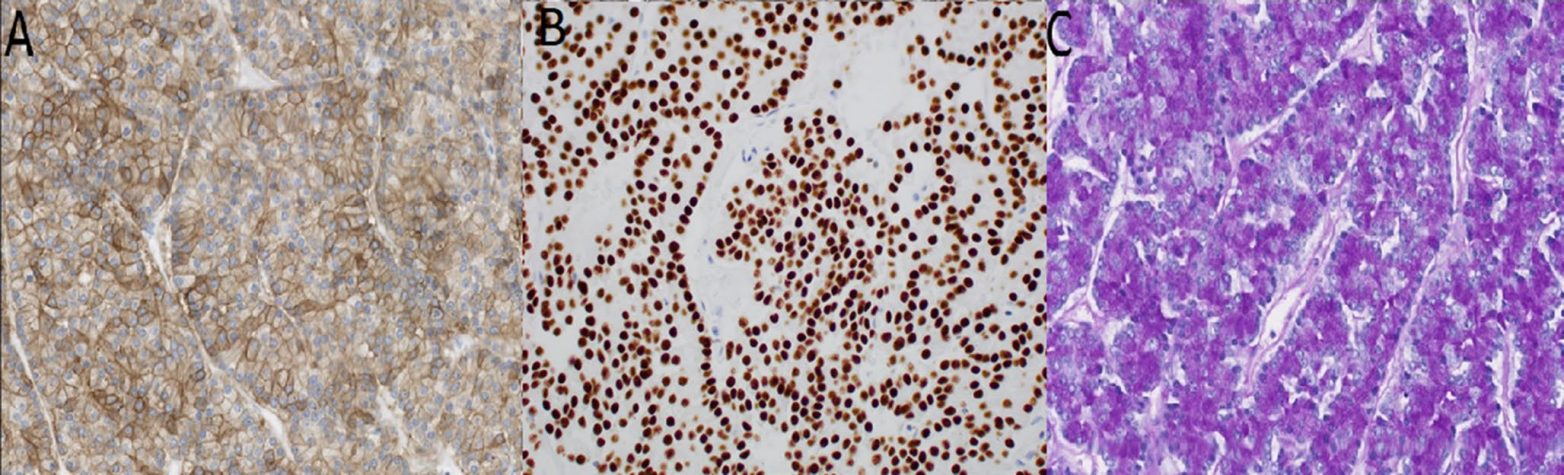
Immunohistochemistry images of tumor from left atrium showing positive DOG1 and NOR1 and focally positive Periodic Acid Schiff with diastase (PASD) staining: (**A**) DOG1 20X, (**B**) NOR1 20X, (**C**) PASD 20X

**Fig. 5:**
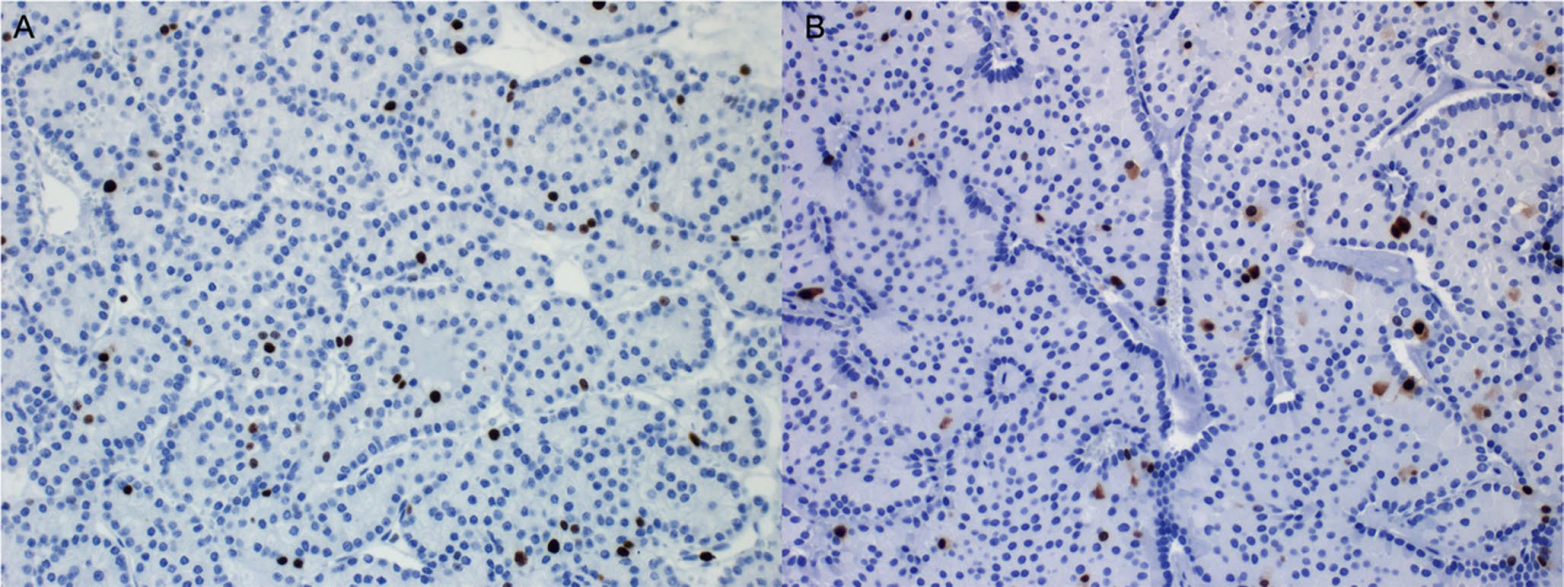
Immunohistochemistry images of Ki-67 showing low Ki-67 of < 1% in both **A**) tumor from left atrium (20X), and **B**) tumor from 4th ventricle of the brain (20X)

**Fig. 6:**
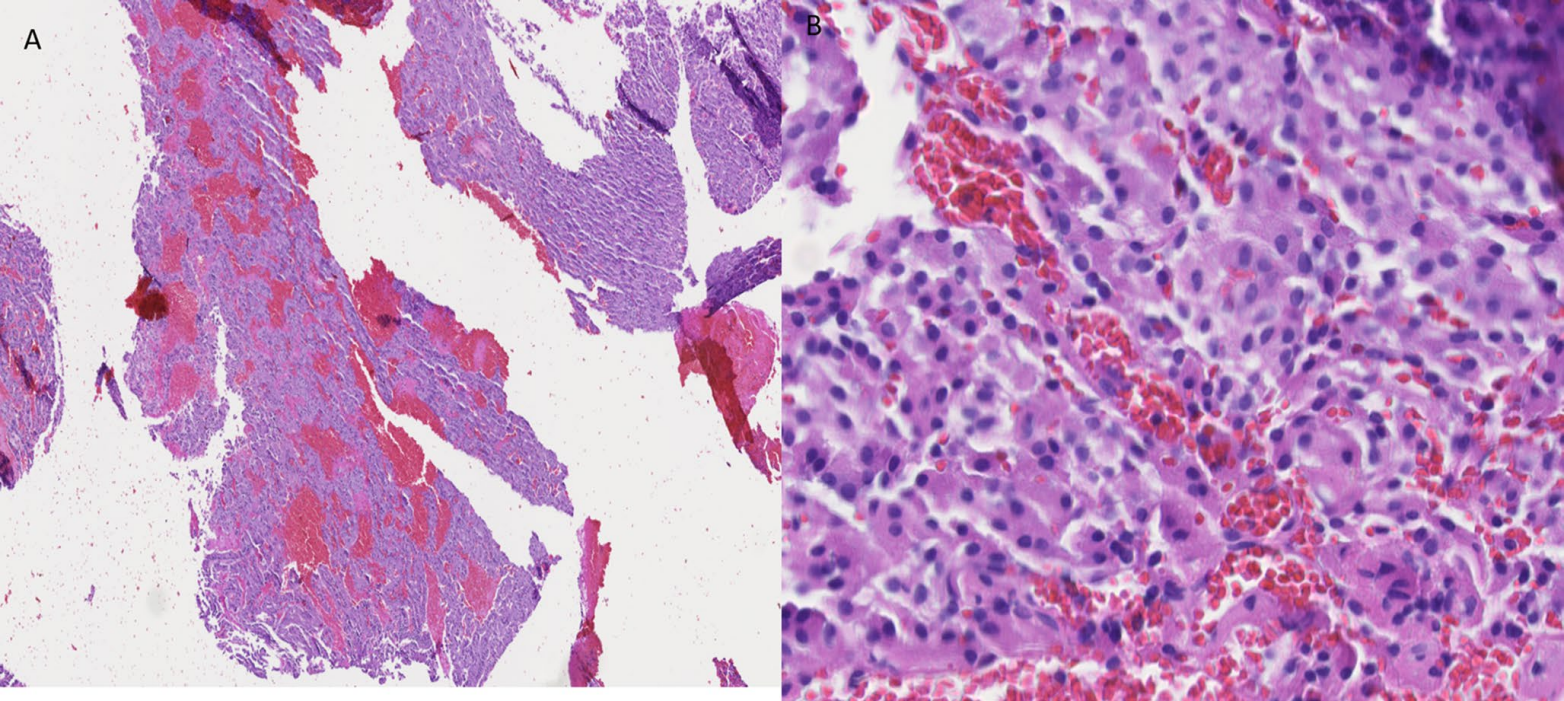
Histology images of the tumor from 4th ventricle of the brain: **A**) Low power magnification, **B**) High power magnification

One week after the removal of the tumor from the left atrium, a pontomedullary intracerebral hemorrhage was discovered (Fig. 2B). The patient underwent suboccipital craniotomy for removal, and histology from the tumor in the 4th ventricle of the brain was consistent with metastatic acinic cell carcinoma, with a similar histomorphology to the mass in the heart (Fig. 6). Ultimately, the patient passed away several months after being found to have brain metastasis.

## Discussion

Acinic cell carcinoma is a relatively rare malignant salivary gland neoplasm [8]. Cases of metastasis of acinic cell carcinoma have been reported to the lungs, cervical lymph nodes, and to the orbit [2, 6, 13–15]. However, as per the authors’ literature review, this is the first case study of metastatic acinic cell carcinoma to the heart.

Although acinic cell carcinoma is considered a low-grade salivary gland neoplasm, it is known to locally recur and metastasize. Case reports of acinic cell carcinoma with distant metastasis have identified lymph node involvement, perineural invasion, and lymphovascular invasion as prognostic predictors that aid in choosing a treatment plan, with the presence of any of these features supporting consideration of adjuvant therapy and more rigorous surveillance [2]. Lymph node metastasis, one of the major prognostic parameters in acinic cell carcinoma, has been reported in up to 10% of cases, despite being considered a low-grade salivary gland malignancy [6].

Currently, there is no specific histologic grading system for acinic cell carcinoma. High-grade transformation of acinic cell carcinoma was first described by Stanley et al. in 1988. It was defined as “areas of dedifferentiated high-grade adenocarcinoma or undifferentiated carcinoma” with associated areas of low-grade acinic cell carcinoma [10]. This and subsequent studies found that increased adverse outcomes are associated with so-called high-grade acinic cell carcinoma [11]. However, the definition of high-grade acinic cell carcinoma has not been consistent [11].

Xu et al. proposed a grading system that includes 4 histologic features: tumor necrosis, mitotic index, fibrosis at invasive front, and tumor borders [11]. In this grading system, acinic cell carcinoma was considered to be low grade for the following parameters: no necrosis or tumor fibrosis, no infiltrative front, and less than or equal to 1 mitoses/ 10 high power fields [11]. Intermediate grade was considered to be 2–4 mitoses/10 high power fields, or infiltrative tumor borders, or fibrosis at an invasive front [11]. High grade was considered to be greater than or equal to 5 mitoses/10 high power fields or tumor necrosis [11]. Using this grading system, high-grade acinic cell carcinomas carried a worse prognosis when compared with low-grade and intermediate-grade acinic cell carcinomas. If we applied this grading system to the case described in this report, the original tumor in the parotid gland would have been considered to the high grade due to the extensive tumor necrosis. This study also identified tumor size, older age, lymphovascular invasion, nuclear anaplasia, necrosis, and advanced pT and pN stages as independent factors for adverse prognosis [11]. The median age of those with low and intermediate grade tumors in this study was 46 years old, while the median age for high grade tumors was 62 years old [11]. The median tumor size for low and intermediate grade tumors was 2.5 cm, and the median size for high grade tumors was 3.0 cm [11].

While there is no formal grading system for acinic cell carcinoma in the current AJCC Cancer Staging System and WHO classification of salivary glands, there is the inclusion of the presence or absence of high-grade transformation, which is defined as marked cytologic atypia, histologically distinct areas, atypical mitoses, increased mitotic activity, and tumor necrosis [12]. In this case, the primary parotid gland tumor possessed high-grade features in the form of significant tumor necrosis, although it possessed a relatively low mitotic rate of 2/2 mm^2^. Thus, histologic grading of the parotid gland tumor may have predicted an adverse prognosis.

Khan et al. conducted a study utilizing data from the Surveillance, Epidemiology, and End Results (SEER) database to identify risk factors that affect survival in patients with acinic cell carcinoma [12]. This study graded tumors as well-differentiated, moderately differentiated, poorly differentiated, and undifferentiated. The study found that the variable with the highest hazard ratio for death compared to the other variables is high tumor grade, as defined by the criteria for high-grade transformation in the current AJCC Cancer Staging System [12]. Thus, this study highlights the significant negative impact of histologic grade on prognosis for patients with acinic cell carcinoma.

Poorly differentiated and high-grade variants of acinic cell carcinoma are known to have increased rates of recurrence and metastasis, usually via hematogenous spread [9]. These high-grade and poorly differentiated variants have a reported recurrence rate of about 35% with a trend for late recurrence, going up to 30 years after initial presentation [13]. Spencer et al. describe a woman who experienced multiple late recurrences of acinic cell carcinoma, with lung and intracranial metastasis occurring 32 years after the initial diagnosis [14]. In a retrospective study with 2362 cases of acinic cell carcinoma of the parotid gland, both high histologic grade and regional metastasis, discussed above, as well as advanced tumor stage, tumor size greater than 3 cm, and age over 70 years old, were all linked to a worse prognosis [16].

Studies have also identified molecular alterations with potential for prognostic implications. The genes most altered in acinic cell carcinoma of the salivary gland are *CDKN2A*, *PTEN*, and *TP53*. Dogan et al. showed a statistically significant worse prognosis in those patients with *CDKN2A/B* alterations, with associated higher-grade morphology and increased risk of distant metastasis [18]. A study demonstrated that over 90% of acinic cell carcinoma cases contain translocation t(4;9) (q13; q31), resulting in the upregulation of nuclear receptor subfamily 4 group member 3 (*NRA4A3*) [7, 17]. This transcription factor can be detected using an antibody to neuron-derived orphan receptor 1 (NOR-1), which showed strong nuclear staining in our case. However, currently there are no available therapies targeting this molecular alteration.

In our case, the patient’s metastatic acinic cell carcinoma was found to harbor a *BRAF* mutation, which occurs in approximately 4–5% of all acinic cell carcinomas. This molecular alteration offers the opportunity to use a *BRAF* inhibitor for treatment; however, the prognostic or therapeutic value of *BRAF* mutations has not been established in acinic cell carcinoma [18]. The 10-year survival of acinic cell carcinoma drops from 99.15% (local disease only) to 31.52% with distant metastasis [19]. Thus, distant metastasis of acinic cell carcinoma is a significant poor prognostic factor for this disease.

Due to the rarity of metastatic salivary gland cancers, there are no large clinical trials to determine the optimal treatment. Vidyadhara et al. detail a case of simultaneous metastasis of acinic cell carcinoma to the lymph nodes, lungs, and spine, and suggest the importance of postoperative radiotherapy if there is incomplete resection of the primary tumor [8]. A study of 301 patients undergoing excision of major salivary glands for cancer at Memorial Sloan-Kettering between 1985 and 2009 showed 20% of salivary gland carcinomas had distant metastasis with most being to the lung (49%) or bone (40%) [4]. In this study, 16% of acinic cell carcinomas developed distant metastasis.

The heart’s unique lymphatic organization protects it from metastasis. Still, metastatic disease is more common than primary tumors of the heart [20]. Metastases are the most common neoplasms of the heart with an incidence between 1.5 and 20% among autopsies of cancer patients [21, 22]. Tumor spreads to the heart via direct spread, bloodstream, lymphatics, and intracavitary migration through the pulmonary veins. Some tumors have displayed higher rates of metastasis to the heart, such as melanoma and mediastinal primary tumors [20]. Metastasis of salivary gland carcinomas to the heart is particularly rare. A few cases of metastatic epithelial-myoepithelial carcinoma of the parotid gland to the heart have been reported [23]. Buchanan et al. describe a case of a woman with metastasis of epithelial myoepithelial carcinoma of the parotid gland to the right heart, which was identified by transthoracic echocardiography [23]. Kishida et al. describe a case of metastatic mucoepidermoid carcinoma to the heart [24]. Foglietta et al. present a case of polymorphous adenocarcinoma of the minor salivary glands with metastasis to the right ventricle [21]. Table 1 provides a summary of prior literature describing salivary gland carcinoma metastasis to the heart.

**Table 1:** Summary of prior literature on salivary gland carcinoma metastasis to the heart

Study	Primary Type of Carcinoma	Description of Case	Cardiac Tissue Involved	Metastasis Diagnosis Method
Buchanan et al. (2022) [23]	Epithelial-myoepithelial carcinoma of the parotid gland	59 y.o. female with metastasis to the right ventricle with reduction of right ventricular systolic function.	Myocardium	Imaging only
Kishida et al. (2022) [24]	Mucoepidermoid carcinoma of the parotid gland	30 y.o. male with cardiac tamponade secondary metastasis to the pericardium of inferior heart.	Pericardium	Histology
Foglietta et al. (2021) [21]	Polymorphous adenocarcinoma of the minor salivary glands	70 y.o. female with metastasis to the right ventricle.	Myocardium	Imaging only
Demirozu et al. (2012) [25]	Adenocarcinoma of the parotid gland	51 y.o. male with metastasis to the right ventricular wall and ventricular septum.	Myocardium	Histology
Barbetakis et al. (2003) [26]	Adenocarcinoma of the parotid gland	61 y.o. female with cardiac tamponade secondary to metastasis to the pericardium.	Pericardium	Cytology
Sulkes et al. (1982) [27]	Adenocarcinoma of the parotid gland	46 y.o. female with pericardial effusion secondary to metastasis to the pericardium.	Pericardium	Histology
Becker et al. (1975) [28]	Mucoepidermoid carcinoma of the parotid gland	39 y.o. female with pericardial effusions secondary to metastasis to the pericardium.	Pericardium	Cytology

To our knowledge, this case report is the only published account of a case of acinic cell carcinoma metastasis to the heart. Due to the significant prognostic significance of distant metastasis in salivary gland carcinomas, this is a unique case of a patient that presented with metastasis to the heart 8 years after initial diagnosis. Because of the rarity of these cases, it is important to highlight the disease course in cases of salivary gland carcinoma with distant metastasis.

In conclusion, conventional acinic cell carcinoma is typically of low aggression; however, in cases of acinic cell carcinoma with high-grade features, such as tumor necrosis or high mitotic index, there is increased risk of distant metastasis. Definition of high-grade features of acinic cell carcinoma can help to stratify patient’s risk to identify patients more likely to have distant metastasis.

## Data Availability

No datasets were generated or analysed during the current study.

## Code Availability

No software application or custom code has been used in the preparation of this case study.
